# Mithramycin A Alleviates Osteoarthritic Cartilage Destruction by Inhibiting HIF-2α Expression

**DOI:** 10.3390/ijms19051411

**Published:** 2018-05-09

**Authors:** Moon-Chang Choi, Woo Hee Choi

**Affiliations:** 1 School of Life Sciences, Gwangju Institute of Science and Technology, Gwangju 61005, Korea; choist777@chosun.ac.kr; 2 Department of Biomedical Science, Chosun University, Gwangju 61452, Korea

**Keywords:** Mithramycin A, osteoarthritis, HIF-2α, SP1, NF-κB

## Abstract

Osteoarthritis (OA) is the most common and increasing joint disease worldwide. Current treatment for OA is limited to control of symptoms. The purpose of this study was to determine the effect of specificity protein 1 (SP1) inhibitor Mithramycin A (MitA) on chondrocyte catabolism and OA pathogenesis and to explore the underlying molecular mechanisms involving SP1 and other key factors that are critical for OA. Here, we show that MitA markedly inhibited expressions of matrix-degrading enzymes induced by pro-inflammatory cytokine interleukin-1β (IL-1β) in mouse primary chondrocytes. Intra-articular injection of MitA into mouse knee joint alleviated OA cartilage destruction induced by surgical destabilization of the medial meniscus (DMM). However, modulation of SP1 level in chondrocyte and mouse cartilage did not alter catabolic gene expression or cartilage integrity, respectively. Instead, MitA significantly impaired the expression of HIF-2α known to be critical for OA pathogenesis. Such reduction in expression of HIF-2α by MitA was caused by inhibition of NF-κB activation, at least in part. These results suggest that MitA can alleviate OA pathogenesis by suppressing NF-κB-HIF-2α pathway, thus providing insight into therapeutic strategy for OA.

## 1. Introduction

Cartilage tissue in the knee joint undergoes degeneration in response to prolonged damage, mechanical stress, and synovial inflammation, resulting in osteoarthritis (OA), an age-related disease and the most common form of arthritis [1]. OA is a challenging disease due to limited treatment options. Most pharmacologic therapies for OA are focused on control of symptoms, pain, and disability. There is no established therapy to prevent progressive cartilage loss in OA joints, to date [2]. OA is characterized by initial loss of proteoglycan in cartilage, changes in subchondral bone, and osteophyte formation at edges of joints [1,3,4]. In normal cartilage, cartilage homeostasis is maintained by optimal balance of anabolic-catabolic signaling in chondrocytes [5,6]. In degenerative cartilage, catabolic factors such as matrix metalloproteinases (MMPs) and aggrecanases (ADAMTSs) are highly induced in chondrocytes, leading to degradation of the extracellular matrix (ECM). In particular, MMP3, MMP13, and ADAMTS5 play critical roles in OA pathogenesis [7,8,9].

Pro-inflammatory cytokine interleukin 1β (IL-1β), produced by autocrine and paracrine mechanisms in OA knee joints, is known to be associated with OA cartilage destruction [10,11,12,13,14,15]. In chondrocytes, IL-1β stimulates the activation of NF-κB and MAP kinase and the expression of several OA-associated key factors, including ZIP8 and HIF-2α [16,17,18,19]. For example, IL-1β upregulates the expression of HIF-2α in chondrocytes, leading to transcriptional induction of MMPs [16,18]. Genetic or pharmacologic inhibition of these key factors can protect cartilage loss from experimental OA. Thus, suppressing OA-associated factors and matrix-degrading enzymes by pharmacological inhibitors might be a primary strategy to prevent OA progression.

Mithramycin A (MitA) is an aureolic acid-type antineoplastic antibiotic. It has been used to treat testicular cancer [20,21] and Paget’s disease of bone [22,23]. MitA primarily inhibits the binding of specificity protein 1 (SP1) transcription factor to its cognate GC-rich motifs in target promoters [24,25]. Since SP1 plays critical roles in diverse biological contexts, such as neurodegeneration and cancer [26,27], MitA has been extensively used in diseases in which abnormal SP1 expression or activation is present [28,29,30,31,32]. However, functional significance of either SP1 or MitA on OA pathogenesis has not yet been established. A previous study has shown that MitA downregulates the expression of MMP3 and MMP13 in chondrocyte induced by IL-1β [33]. Whether MitA alleviates OA pathogenesis in vivo or how MitA suppresses catabolic gene expression has not been elucidated, yet.

Here, we examined the effects of MitA in cultured chondrocytes and OA cartilages. We found that MitA alleviates osteoarthritic cartilage lesion and strongly represses catabolic factor expression independent of canonical inhibitory mechanism toward SP1. We identify NF-κB-HIF-2α signaling as a major pathway responsible for MitA-suppressed catabolic gene expression. Overall, our findings might provide a potential drug candidate for OA as a treatment option.

## 2. Results

### 2.1. Mithramycin A (MitA) Inhibits Induction of MMPs by IL-1β in Mouse Primary Chondrocytes

**Figure 1:**
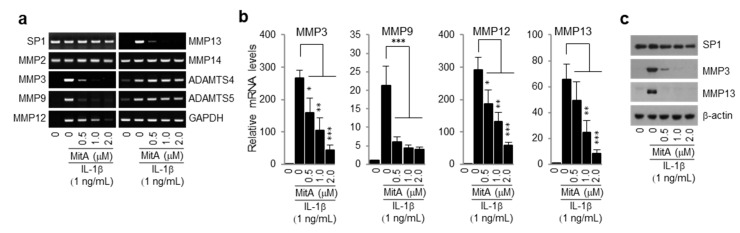
Mithramycin A (MitA) inhibits IL-1β-induced expression of matrix-degrading enzymes in mouse primary chondrocytes. (**a**,**b**) mRNA expression levels of indicated genes were detected in chondrocytes treated with IL-1β alone or treated with IL-1β and MitA for 24 h. Representative images of RT-PCR (**a**) and quantification using real-time PCR (**b**) are shown (*n* = 5). Values are means ± SEM (* *p* < 0.05, ** *p* < 0.01, *** *p* < 0.001); (**c**) Protein levels of SP1 (cellular) and MMP3/MMP13 (extracellular) were determined by Western blotting.

It is known that activation of catabolic signaling pathways in chondrocytes promotes cartilage degeneration [7,9,34]. To investigate whether MitA could regulate the expression of matrix degrading enzymes in OA chondrocytes, expression levels of MMPs and ADAMTSs were determined in mouse primary chondrocytes treated with IL-1β alone or with IL-1β and MitA. The result showed that MitA significantly abrogated mRNA inductions of MMP3, MMP9, MMP12, and MMP13 by IL-1β in a dose-dependent manner (Figure 1a). In contrast, mRNA expression level of MMP2 or MMP14 was not significantly changed by either IL-1β or MitA. Inductions of ADAMTS4 and ADAMTS5 mRNAs by IL-1β were not repressed in MitA-treated chondrocytes. qPCR analysis confirmed the suppression of MMP3, MMP9, MMP12, and MMP13 mRNAs by MitA (Figure 1b). Consistently, levels of secreted extracellular MMP3 and MMP13 proteins were markedly decreased in MitA-treated chondrocytes (Figure 1c). SP1 expression was not altered by MitA treatment (Figure 1a,c). These results indicate that MitA can potently inhibit IL-1β-induced catabolic gene expression.

### 2.2. MitA Administration to Mouse Knee Joint Delays the Progression of Experimental OA

**Figure 2:**
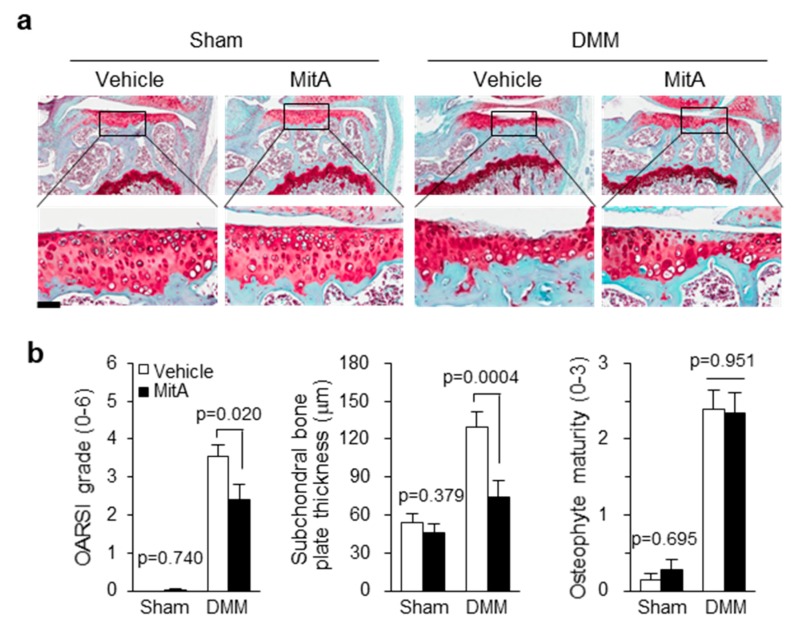
MitA injection to mouse knee joints alleviates post-traumatic OA. 10-week-old male mice operated by DMM surgery were treated with MitA through intra-articular (IA) injection. Mice were harvested 8 weeks later after surgical destabilization of the medial meniscus (DMM). Representative images of safranin-O staining (**a**) and OA-related parameters including OARSI grade, sclerosis, and osteophyte maturity are shown (**b**; *n* = 12). Values are means ± SEM. Scale bar: 50 μM.

We next investigated whether MitA could inhibit traumatic OA progression in vivo. To induce traumatic OA, 10-week-old male mice were subjected to surgical destabilization of the medial meniscus (DMM). At 10 days after DMM operation, the first treatment of MitA was achieved by intra-articular (IA) injection. MitA was then injected at 10-day intervals. Cartilages collected 8 weeks after DMM surgery were stained with safranin-O to determine cartilage loss, subchondral bone plate thickness, and osteophyte formation. As shown in Figure 2a, in sham knee joints, cartilage integrities were comparable between vehicle- and MitA-treated groups. Importantly, MitA treatment alleviated DMM-induced cartilage erosion compared to vehicle-treated DMM cartilages based on safranin-O staining and OARSI scoring (Figure 2a,b). Supporting this result, MitA-treated DMM mice also showed less sclerosis than vehicle-treated DMM mice, suggesting that post-traumatic OA progression was indeed decelerated by MitA injection. In contrast, maturity of osteophyte was not changed by MitA treatment (Figure 2b). These results indicate that MitA can alleviate osteoarthritic cartilage lesions.

### 2.3. Effect of MitA on OA is Independent of SP1 Level

**Figure 3:**
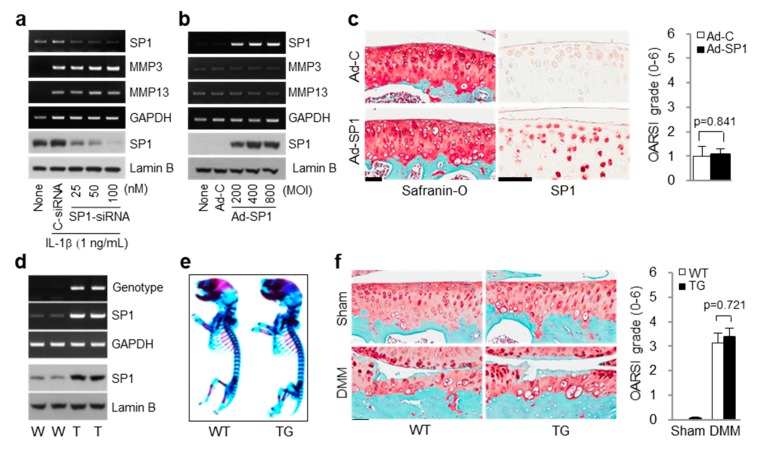
SP1 is not required for MMP induction or OA. (**a**) Effects of SP1 knockdown using SP1 siRNA on MMP expression. mRNA and protein levels of indicated genes were determined by RT-PCR and Western blotting, respectively; (**b**) Overexpression of SP1 in chondrocytes does not induce expression of MMP3 or MMP13. mRNA levels in chondrocytes infected with adenoviral vector coding SP1 (Ad-SP1) with indicated MOI were determined by RT-PCR. Adenoviral vector lacking an insert (Ad-C) was used as a control (800 MOI); (**c**) Representative images of safranin-O staining, SP1 immunostaining, and OARSI scoring of cartilages from mice injected with Ad-C or Ad-SP1 (*n* = 5). Values are means ± SEM. Scale bar: 50 μM; (**d**–**f**) Effects of SP1 transgenic (TG) mice on OA; (**d**) Characterization of Col2a1-SP1 TG mice and wild type (WT) littermates; (**e**) Skeletal staining at embryonic day 18.5 (E18.5) of SP1 TG mice and their WT littermates; (**f**) Representative images of safranin-O staining and OARSI scoring of cartilages collected from WT and SP1 TG mice operated by DMM surgery (*n* ≥ 7). Values are means ± SEM. Scale bar: 50 μM.

Because SP1 transcription factor is a primary target of MitA, our initial hypothesis was that MitA might inhibit SP1 to suppress chondrocyte catabolism. To test this hypothesis, we modulated the level of SP1 in chondrocytes by using siRNA knockdown (KD) or adenoviral-mediated overexpression system to determine catabolic gene expression. Unexpectedly, SP1 KD did not reduce induction of MMP3 or MMP13 by IL-1β (Figure 3a). Overexpression of SP1 using Ad-SP1 did not increase expression of MMP3 or MMP13 in chondrocytes (Figure 3b). Consistent with these in vitro results, Ad-SP1 infection to mouse knee joint showed less effect on cartilage destruction (Figure 3c). To ascertain cartilage-specific functions of SP1 in OA, we generated cartilage-specific SP1 transgenic (TG) mice (Col2a1-SP1). Primary cultured chondrocytes from SP1 TG mice exhibited upregulation of SP1 at mRNA and protein levels compared to those from wild type (WT) littermates without detectable defects in skeletal development (Figure 3d,e). In vivo significance of SP1 overexpression in cartilage tissue was evaluated by DMM surgery for 8 weeks using 10-week-old male mice. As shown in Figure 3f, cartilage erosion induced by experimental OA was comparable between WT and SP1 TG mice. These results suggest that SP1 alone is insufficient to affect OA pathogenesis, suggesting that MitA might inhibit catabolic gene expression via SP1-independent mechanism.

### 2.4. MitA Suppresses HIF-2α Induction by Inhibiting NF-κB Activation

**Figure 4:**
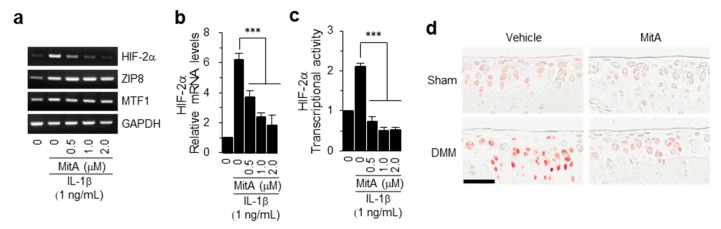
MitA inhibits expression of HIF-2α in chondrocyte and OA cartilage tissue. (**a**,**b**) Expression of HIF-2α is repressed in MitA-treated chondrocytes. Representative images of RT-PCR (**a**) and quantification using real-time PCR (**b**) are shown (*n* ≥ 5). Values are means ± SEM (*** *p* < 0.001); (**c**) Effect of MitA on HIF-2α activity. Transcriptional activity of HIF-2α was assessed using hypoxia-response element (HRE) reporter assay in MitA-treated chondrocytes (*n* = 4). Values are means ± SEM (*** *p* < 0.001); (**d**) HIF-2α immunostaining of cartilage tissue collected from mice after DMM surgery and/or MitA IA injection. Scale bar: 50 μM.

Several key regulators associated with OA pathogenesis have been identified, including HIF-2α, ZIP8, and MTF1. Activation of these genes is sufficient to promote chondrocyte catabolism and OA development [16,17,18]. We thus investigated whether MitA could inhibit expression levels of these factors in chondrocytes. Our results revealed that HIF-2α expression was markedly suppressed in MitA-treated chondrocytes (Figure 4a,b). Consistently, HIF-2α reporter activity was also decreased in MitA-treated chondrocytes (Figure 4c). This might be due to reduction of HIF-2α expression. In contrast, expression of ZIP8 or MTF1 was not affected by MitA treatment (Figure 4a). Changes in HIF-2α expression after MitA treatment were also evaluated in DMM-operated cartilage tissues (Figure 4d). In contrast to strong induction of HIF-2α by DMM surgery, expression of HIF-2α in cartilages from MitA-treated knee joints was found to be reduced. These in vitro and in vivo findings indicate that MitA can suppress HIF-2α expression.

**Figure 5:**
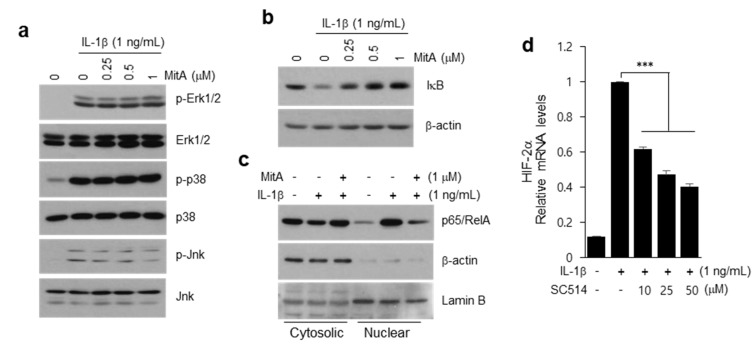
IL-1β-induced activation of NF-κB, but not MAP kinases, is inhibited by MitA in chondrocytes. (**a**–**c**) chondrocytes were pre-treated with MitA for 24 h and subsequently treated with IL-1β for 10 min. (**a**) MitA does not inhibit activation of MAP kinases. Activations of MAP3 kinases, Erk, p38, and Jnk were determined by Western blotting with phospho-antibodies; (**b**,**c**) MitA suppresses degradation of IκB and translocation of NF-κB; (**b**) Degradation of IκB was determined by Western blotting; (**c**) Nuclear translocation of p65/RelA was determined by subcellular fractionation and Western blotting. β-actin and Lamin B were used as markers of cytoplasmic and nuclear fractions, respectively; (**d**) Inhibition of HIF-2α expression by SC514, an IKKβ inhibitor, in IL-1β-treated chondrocytes (*n* = 4). Values are means ± SEM (*** *p* < 0.001).

We next examined possible cross-talk of MAPK or NF-κB signaling with MitA-mediated suppression of HIF-2α. MitA treatment did not alter the activation of MAP3 kinases, Erk, p38, or Jnk induced by IL-1β (Figure 5a), consistent with a previous report [33]. Interestingly, MitA suppressed NF-κB activation, namely degradation of IκB, by IL-1β (Figure 5b). Supporting this finding, MitA also reduced nuclear translocation of p65/RelA induced by IL-1β (Figure 5c). NF-κB is known to promote catabolic gene expression program and OA pathogenesis [35,36]. It is also involved in up-regulation of HIF-2α [16,18]. We confirmed that inhibition of NF-κB by SC514 reduces IL-1β-induced upregulation of HIF-2α in chondrocytes (Figure 5d). Together, these results suggest that the inhibitory action of MitA on catabolic gene expression is through suppression of the NF-κB-HIF-2α axis.

## 3. Discussion

In this study, we provided evidence that MitA could alleviate chondrocyte catabolism and OA pathogenesis by inhibiting HIF-2α expression. Induction of catabolic factors is a key feature that destroys the balance of cartilage homeostasis. Thus, blockage of catabolic signaling pathway has been recognized as a treatment strategy for OA [37,38,39]. In this regard, our findings provide a potential drug candidate for targeting chondrocyte catabolism in OA. Although reduction of MMP3 and MMP13 by MitA has been previously reported in human chondrocytes [33], in this study, we further explored the effect of MitA on OA cartilage destruction and the underlying molecular mechanism. Our results suggest that MitA might be applicable for therapeutic intervention of OA.

Our results revealed that MitA inhibited the expression of a subset of MMPs, but not the expression of ADAMTSs, in mouse primary chondrocyte. In contrast, a previous study has shown partial reduction of ADAMTS4 by MitA and SP1-dependent regulation of ADAMTS4 in human chondrocyte [40]. Such discrepancy in ADAMTS4 expression by MitA requires further investigation. Because KO of MMP3, MMP13, or ADAMTS5 is sufficient to abrogate cartilage destruction [7,8,9], significant suppression of MMP3 and MMP13 by MitA treatment in chondrocytes supports our hypothesis that MitA might attenuate OA cartilage destruction. Incomplete suppression of cartilage lesions in MitA-treated DMM cartilages leaves open two possibilities: little effect of MitA on expression of ADAMTS5; and/or limitation of MitA on inflammation-associated OA, but not mechanical stress-associated. In the latter case, mechanical stress also activates NF-κB, which contributes to OA development [41,42,43,44]. Whether MitA suppresses mechanical stress-induced NF-κB activation and OA pathogenesis is interesting. It requires further studies.

Our results also demonstrated that anti-catabolic effects of MitA were not mediated by inhibition of SP1, but through HIF-2α inhibition. For gain-of-function SP1, cartilage-specific TG mouse and adenoviral-mediated overexpression were used in this study. Our results from both methods revealed that forced expression of SP1 failed to induce catabolic gene expression or cartilage destruction. This unexpected finding might raise the possibility that SP1 activity, rather than up-regulation, is required to activate catabolic pathways in chondrocytes that express basal abundance of SP1 protein (Figure 1c). However, SP1 depletion using siRNA KD did not reduce expression of MMP3 or MMP13. Furthermore, KD of SP3, another target of MitA [24,45], or double KDs of SP1 and SP3 failed to inhibit IL-1β-induced matrix degrading enzymes either. Collectively, our results suggest that MMP suppression by MitA is independent of its canonical inhibitory function toward SP1/SP3. Further investigation using SP1 knockout mice might be necessary to validate this speculation.

Several reports have shown SP1-independent gene expression after MitA treatment [46,47]. By examining OA-associated genes and signaling pathways, we identified HIF-2α as a factor specifically down-regulated by MitA. Such down-regulation was mediated by NF-κB inactivation. It has been reported that inactivation of either HIF-2α or NF-κB p65 in chondrocytes abrogates the expression of MMPs [16,18,48]. Therefore, our results suggest that MitA might alleviate MMP induction and osteoarthritic cartilage lesion by suppressing NF-κB-HIF-2α signaling, at least in part. How MitA communicates with NF-κB signaling is currently unclear. It is possible that MitA can directly affect the expression and/or activation of genes associated with toll-like receptor (TLR) signaling. Another possibility is that MitA can regulate histone modifications, resulting in dysregulation of NF-κB activation. Besides DNA, MitA can also bind to core histones present in chromatin and inhibit histone H3 acetylation to regulate gene expressions [47]. Moreover, MitA can inhibit a subset of class I histone deacetylase expression [49]. More work might be required to understand the underlying mechanism(s) by which MitA inhibits NF-κB and whether MitA also targets other key factors critical for OA pathogenesis.

## 4. Materials and Methods

### 4.1. Chondrocyte Culture and MitA Treatment

Mouse primary articular chondrocytes were isolated from femoral condyles and tibial plateaus of five-day-old mice followed by digestion with 0.2% collagenase (Sigma, St. Louis, MO, USA) [48]. Cells were cultured in Dulbecco’s Modified Eagle’s Medium (DMEM) supplemented with 10% fetal bovine serum (FBS) and antibiotics. They were then harvested within a week without subculture. MitA (Sigma) was pre-treated for one hour before IL-1β treatment (1 ng/mL; Genescript, Piscataway, NJ, USA) unless otherwise indicated.

### 4.2. Adenoviruses, Infection of Chondrocytes, and Intra-Articular (IA) Injection in Mice

Adenovirus lacking an insert (Ad-C) and expressing mouse SP1 (Ad-SP1) was purchased from Vector Biolabs. Adenoviral vectors amplified in HEK293 cells were purified by CsCl density-gradient centrifugation and dialyzed in GTS buffer (20 mM Tris, pH 7.5, 25 mM NaCl, 2.5% glycerol) [50]. Adeno-X rapid titer kit (Clontech, Palo Alto, CA, USA) was used to determine viral titer. Chondrocytes were infected with Ad-SP1 for two hours at indicated multiplicities of infection (MOI), washed with PBS, and cultured for 36 h. For IA injection to mouse knee joints, 10-week-old male mice were injected with Ad-SP1 (1 × 10^9^ plaque-forming units in a total volume of 10 μL) once weekly for 3 weeks. They were sacrificed at 3 weeks after the first IA injection.

### 4.3. RNA Analysis

Total RNA was extracted from chondrocytes using TRI reagent (Molecular Research Center, Cincinnati, OH, USA). cDNA synthesis was conducted using oligo dT primer and Improm II reverse transcription system (Promega, ‎Fitchburg, WI, USA‎) [51]. PCR primers and conditions are summarized in Supplementary Table S1. Quantitative RT-PCR (qRT-PCR) was conducted in a CFX Connect Real-Time PCR Detection System (Bio-Rad, Hercules, CA, USA) using SYBR Premix Ex Taq (TaKaRa, Singa, Japan).

### 4.4. Western Blot Analysis and Subcellular Fractionation

Whole cell lysates of chondrocytes were prepared in lysis buffer (50 mM Tris (pH 7.5), 150 mM NaCl, 1% NP-40, 0.5% sodium deoxycholate, 1 mM EDTA) containing protease inhibitor cocktail (Roche, Basel, Switzerland) and phosphatase inhibitor cocktail (Roche) [52]. Subcellular fractionation was conducted with NE-PER nuclear and cytoplasmic extraction reagents (Thermo Scientific, Waltham, MA, USA) following the manufacturer’s instructions. Protein concentrations were determined using BCA assay (Sigma). Equal amounts of cell lysates were separated on SDS-PAGE gel, transferred onto nitrocellulose membrane, and probed with antibodies of SP1, β-actin, MMP3, MMP13 (Abcam, Cambridge, UK), Lamin B (Santa Cruz Biotechnology, Santa Cruz, CA, USA), p-Erk1/2, Erk1/2, p-p38, p38, p-Jnk, Jnk, p65, or IκB (Cell Signaling Technology, ‎Danvers, MA, USA). Blots were detected using an ECL system (Amersham, Little Chalfont, UK).

### 4.5. siRNA Knockdown (KD) of SP1 and HIF-2α Reporter Assay

For KD study of SP1, chondrocytes were transfected with SP1 siRNA (Ambion, Austin, TX, USA) using Lipofectamine RNAiMAX (Invitrogen, Carlsbad, CA, USA) according to the manufacturer’s instructions. The same concentration of control siRNA was used as negative control. Cells were harvested at 24 h after IL-1β treatment. For HIF-2α reporter activity, HIF-2α luciferase reporter was co-transfected with renilla reporter using Lipofectamine 2000 (Invitrogen). Transfected cells were either left untreated or treated as indicated. Cells were harvested at 24 h post-treatment. Firefly and renilla luciferase activities were measured using dual luciferase reporter assay kit (Promega).

### 4.6. Mice and Experimental OA

Male mice in C57BL/6 background were used for animal experiments. Chondrogenic-specific SP1 transgenic (TG) mice were generated using Col2a1 promoter and enhancer [53] by Marcogen Inc. (Seoul, Korea) using microinjection technique. SP1 TG mice were identified via PCR of tail genotyping using SP1 TG screening primers (sense, 5′-TACAGGGGAGAGGCCATTCA-3′; anti-sense, 5′-TGTATCTTATCATGTGGGAT-3′). Experimental OA was induced by surgical destabilization of the medial meniscus (DMM) [48]. At 10 days after DMM surgery, the first treatment of MitA (0.1 mg/kg) was achieved by IA injection. MitA was then injected at 10-day intervals for total five times until mice were sacrificed at 8 weeks for histological analysis. Animals were maintained under pathogen-free conditions. All experiments were approved by the Committee for the Care and Use of Laboratory Animal in Gwangju Institute of Science and Technology (GIST-2014-20).

### 4.7. Histology, Immunohistochemistry, and Skeletal Staining

Knee joints of mice were fixed in 4% paraformaldehyde, decalcified in 0.5 M EDTA, and embedded in paraffin wax. Paraffin blocks were sectioned at a thickness of 5 μm. To achieve representative sections from the whole cartilage tissue, serial sections were taken every 10–15 slices and 3–4 sections per cartilage were stained with safranin-O. The degree of cartilage destruction was scored by two blind observers using OARSI grading system [54]. Subchondral bone sclerosis was determined by measuring the thickness of the subchondral bone plate. For immunohistochemistry, antigen retrieval was performed by incubating sections at 37 °C for 30 min with 0.05% trypsin in 0.1% CaCl_2_ (pH 7.8). After blocking with 1% BSA in PBS, sections were incubated at 4 °C overnight with primary antibody against SP1 (Abcam) or HIF-2α (Abcam). Sections were then incubated with secondary antibody for 30 min followed by incubation with chromagene AEC (Dako, Cambridge, UK). For skeletal staining, skeletons of whole mice at embryonic day 18.5 were stained with alcian blue and alizarin red. Briefly, whole embryos were skinned, eviscerated, fixed with 95% ethanol for 4 days, and immersed in acetone for 3 days. Samples were stained for 7 days in a solution composed of one volume of 0.3% Alcian blue 8GX in 70% ethanol, one volume of 0.1% Alizarin red S in 95% ethanol, one volume of 100% acetic acid, and 17 volumes of 100% ethanol. Samples were destained with 1% KOH for 48 h and further destained with 20% glycerol containing 1% KOH for 14 days.

### 4.8. Statistical Analysis

Data quantified based on ordinal grading system, such as OARSI grade, were analyzed using non-parametric Mann-Whitney U test. Data of qRT-PCR expressed as relative fold changes were analyzed by one-way analysis of variance (ANOVA) with Tukey’s post hoc test using SPSS 12.0.1 (SPSS, Inc., Chicage, IL, USA). The number of independent experiments or mice is indicated by *n*. Significance was considered at *p* < 0.05.

## Supplementary Materials

Supplementary materials can be found at http://www.mdpi.com/1422-0067/19/5/1411/s1.

## Abbreviations

DMM	Destabilization of medial meniscus
HIF-2α	Hypoxia-inducible factor-2α
IL-1β	Interleukin-1β
MitA	Mithramycin A
MMP	Matrix metalloproteinase
NF-κB	Nuclear factor kappa B
OA	Osteoarthritis
SP1	Specificity protein 1

## References

[1] Goldring M.B., Goldring S.R. (2007). Osteoarthritis. J. Cell. Physiol..

[2] Zhang W., Ouyang H., Dass C.R., Xu J. (2016). Current research on pharmacologic and regenerative therapies for osteoarthritis. Bone Res..

[3] Bian Q., Wang Y.J., Liu S.F., Li Y.P. (2012). Osteoarthritis: Genetic factors, animal models, mechanisms, and therapies. Front. Biosci..

[4] Burr D.B., Gallant M.A. (2012). Bone remodelling in osteoarthritis. Nat. Rev. Rheumatol..

[5] Heinegard D., Saxne T. (2011). The role of the cartilage matrix in osteoarthritis. Nat. Rev. Rheumatol..

[6] Bonnans C., Chou J., Werb Z. (2014). Remodelling the extracellular matrix in development and disease. Nat. Rev. Mol. Cell Biol..

[7] Blom A.B., van Lent P.L., Libregts S., Holthuysen A.E., van der Kraan P.M., van Rooijen N., van den Berg W.B. (2007). Crucial role of macrophages in matrix metalloproteinase-mediated cartilage destruction during experimental osteoarthritis: Involvement of matrix metalloproteinase 3. Arthritis Rheum.

[8] Glasson S.S., Askew R., Sheppard B., Carito B., Blanchet T., Ma H.L., Flannery C.R., Peluso D., Kanki K., Yang Z. (2005). Deletion of active adamts5 prevents cartilage degradation in a murine model of osteoarthritis. Nature.

[9] Little C.B., Barai A., Burkhardt D., Smith S.M., Fosang A.J., Werb Z., Shah M., Thompson E.W. (2009). Matrix metalloproteinase 13-deficient mice are resistant to osteoarthritic cartilage erosion but not chondrocyte hypertrophy or osteophyte development. Arthritis Rheumatol..

[10] Ritchlin C. (2000). Fibroblast biology. Effector signals released by the synovial fibroblast in arthritis. Arthritis Res..

[11] Wang P., Guan P.P., Guo C., Zhu F., Konstantopoulos K., Wang Z.Y. (2013). Fluid shear stress-induced osteoarthritis: Roles of cyclooxygenase-2 and its metabolic products in inducing the expression of proinflammatory cytokines and matrix metalloproteinases. FASEB J..

[12] Towle C.A., Hung H.H., Bonassar L.J., Treadwell B.V., Mangham D.C. (1997). Detection of interleukin-1 in the cartilage of patients with osteoarthritis: A possible autocrine/paracrine role in pathogenesis. Osteoarthr. Cartil..

[13] Kapoor M., Martel-Pelletier J., Lajeunesse D., Pelletier J.P., Fahmi H. (2011). Role of proinflammatory cytokines in the pathophysiology of osteoarthritis. Nat. Rev. Rheumatol..

[14] Fernandes J.C., Martel-Pelletier J., Pelletier J.P. (2002). The role of cytokines in osteoarthritis pathophysiology. Biorheology.

[15] Arend W.P., Dayer J.M. (1995). Inhibition of the production and effects of interleukin-1 and tumor necrosis factor alpha in rheumatoid arthritis. Arthritis Rheumatol..

[16] Saito T., Fukai A., Mabuchi A., Ikeda T., Yano F., Ohba S., Nishida N., Akune T., Yoshimura N., Nakagawa T. (2010). Transcriptional regulation of endochondral ossification by hif-2alpha during skeletal growth and osteoarthritis development. Nat. Med..

[17] Kim J.H., Jeon J., Shin M., Won Y., Lee M., Kwak J.S., Lee G., Rhee J., Ryu J.H., Chun C.H. (2014). Regulation of the catabolic cascade in osteoarthritis by the zinc-ZIP8-MTF1 axis. Cell.

[18] Yang S., Kim J., Ryu J.H., Oh H., Chun C.H., Kim B.J., Min B.H., Chun J.S. (2010). Hypoxia-inducible factor-2alpha is a catabolic regulator of osteoarthritic cartilage destruction. Nat. Med..

[19] Liacini A., Sylvester J., Li W.Q., Zafarullah M. (2002). Inhibition of interleukin-1-stimulated MAP kinases, activating protein-1 (AP-1) and nuclear factor kappa B (NF-kappa B) transcription factors down-regulates matrix metalloproteinase gene expression in articular chondrocytes. Matrix Biol..

[20] Kennedy B.J., Torkelson J.L. (1995). Long-term follow-up of stage iii testicular carcinoma treated with mithramycin (plicamycin). Med. Pediatr. Oncol..

[21] Brown J.H., Kennedy B.J. (1965). Mithramycin in the treatment of disseminated testicular neoplasms. N. Engl. J. Med..

[22] Remsing L.L., Bahadori H.R., Carbone G.M., McGuffie E.M., Catapano C.V., Rohr J. (2003). Inhibition of c-src transcription by mithramycin: Structure-activity relationships of biosynthetically produced mithramycin analogues using the c-src promoter as target. Biochemistry.

[23] Hall T.J., Schaeublin M., Chambers T.J. (1993). The majority of osteoclasts require mrna and protein synthesis for bone resorption in vitro. Biochem. Biophys. Res. Commun..

[24] Ray R., Snyder R.C., Thomas S., Koller C.A., Miller D.M. (1989). Mithramycin blocks protein binding and function of the sv40 early promoter. J. Clin. Investig..

[25] Lombo F., Menendez N., Salas J.A., Mendez C. (2006). The aureolic acid family of antitumor compounds: Structure, mode of action, biosynthesis, and novel derivatives. Appl. Microbiol. Biotechnol..

[26] Qiu Z., Norflus F., Singh B., Swindell M.K., Buzescu R., Bejarano M., Chopra R., Zucker B., Benn C.L., DiRocco D.P. (2006). Sp1 is up-regulated in cellular and transgenic models of huntington disease, and its reduction is neuroprotective. J. Biol. Chem..

[27] Beishline K., Azizkhan-Clifford J. (2015). Sp1 and the ‘hallmarks of cancer’. FEBS J..

[28] Sleiman S.F., Langley B.C., Basso M., Berlin J., Xia L., Payappilly J.B., Kharel M.K., Guo H., Marsh J.L., Thompson L.M. (2011). Mithramycin is a gene-selective sp1 inhibitor that identifies a biological intersection between cancer and neurodegeneration. J. Neurosci..

[29] Choi E.S., Nam J.S., Jung J.Y., Cho N.P., Cho S.D. (2014). Modulation of specificity protein 1 by mithramycin a as a novel therapeutic strategy for cervical cancer. Sci. Rep..

[30] Ryu H., Lee J., Hagerty S.W., Soh B.Y., McAlpin S.E., Cormier K.A., Smith K.M., Ferrante R.J. (2006). Eset/setdb1 gene expression and histone h3 (k9) trimethylation in huntington’s disease. Proc. Natl. Acad. Sci. USA.

[31] Deacon K., Onion D., Kumari R., Watson S.A., Knox A.J. (2012). Elevated SP-1 transcription factor expression and activity drives basal and hypoxia-induced vascular endothelial growth factor (VEGF) expression in non-small cell lung cancer. J. Biol. Chem..

[32] Osada N., Kosuge Y., Ishige K., Ito Y. (2013). Mithramycin, an agent for developing new therapeutic drugs for neurodegenerative diseases. J. Pharmacol. Sci..

[33] Liacini A., Sylvester J., Li W.Q., Zafarullah M. (2005). Mithramycin downregulates proinflammatory cytokine-induced matrix metalloproteinase gene expression in articular chondrocytes. Arthritis Res. Ther..

[34] Goldring M.B., Otero M., Plumb D.A., Dragomir C., Favero M., El Hachem K., Hashimoto K., Roach H.I., Olivotto E., Borzi R.M. (2011). Roles of inflammatory and anabolic cytokines in cartilage metabolism: Signals and multiple effectors converge upon MMP-13 regulation in osteoarthritis. Eur. Cell Mater..

[35] Rigoglou S., Papavassiliou A.G. (2013). The NF-kappab signalling pathway in osteoarthritis. Int. J. Biochem. Cell Biol..

[36] Roman-Blas J.A., Jimenez S.A. (2006). Nf-kappab as a potential therapeutic target in osteoarthritis and rheumatoid arthritis. Osteoarthr. Cartil..

[37] Xu X., Lv H., Li X., Su H., Zhang X., Yang J. (2017). Danshen attenuates osteoarthritis-related cartilage degeneration through inhibition of nf-kappab signaling pathway in vivo and in vitro. Biochem. Cell Biol..

[38] Yao N., Chen N., Xu X., Sun D., Liu W., Li G., Bi X., Li S., Chen Z., Chen G. (2017). Protective effect of shenmai injection on knee articular cartilage of osteoarthritic rabbits and IL-1beta-stimulated human chondrocytes. Exp. Ther. Med..

[39] Wang K., Li Y., Han R., Cai G., He C., Wang G., Jia D. (2017). T140 blocks the SDF-1/CXCR4 signaling pathway and prevents cartilage degeneration in an osteoarthritis disease model. PLoS ONE.

[40] Sylvester J., Ahmad R., Zafarullah M. (2013). Role of sp1 transcription factor in interleukin-1-induced adamts-4 (aggrecanase-1) gene expression in human articular chondrocytes. Rheumatol. Int..

[41] Marcu K.B., Otero M., Olivotto E., Borzi R.M., Goldring M.B. (2010). NF-kappab signaling: Multiple angles to target OA. Curr. Drug Targets.

[42] Madhavan S., Anghelina M., Sjostrom D., Dossumbekova A., Guttridge D.C., Agarwal S. (2007). Biomechanical signals suppress tak1 activation to inhibit nf-kappab transcriptional activation in fibrochondrocytes. J. Immunol..

[43] Pulai J.I., Chen H., Im H.J., Kumar S., Hanning C., Hegde P.S., Loeser R.F. (2005). Nf-kappa b mediates the stimulation of cytokine and chemokine expression by human articular chondrocytes in response to fibronectin fragments. J. Immunol..

[44] Knobloch T.J., Madhavan S., Nam J., Agarwal S., Agarwal S. (2008). Regulation of chondrocytic gene expression by biomechanical signals. Crit. Rev. Eukaryot. Gene Expr..

[45] Wilson A.J., Chueh A.C., Togel L., Corner G.A., Ahmed N., Goel S., Byun D.S., Nasser S., Houston M.A., Jhawer M. (2010). Apoptotic sensitivity of colon cancer cells to histone deacetylase inhibitors is mediated by an sp1/sp3-activated transcriptional program involving immediate-early gene induction. Cancer Res..

[46] Otjacques E., Binsfeld M., Rocks N., Blacher S., Vanderkerken K., Noel A., Beguin Y., Cataldo D., Caers J. (2013). Mithramycin exerts an anti-myeloma effect and displays anti-angiogenic effects through up-regulation of anti-angiogenic factors. PLoS ONE.

[47] Banerjee A., Sanyal S., Kulkarni K.K., Jana K., Roy S., Das C., Dasgupta D. (2014). Anticancer drug mithramycin interacts with core histones: An additional mode of action of the DNA groove binder. FEBS Open Bio.

[48] Choi M.C., MaruYama T., Chun C.H., Park Y. (2018). Alleviation of murine osteoarthritis by cartilage-specific deletion of ikappabzeta. Arthritis Rheumatol..

[49] Sleiman S.F., Berlin J., Basso M., Karuppagounder S.S., Rohr J., Ratan R.R. (2011). Histone deacetylase inhibitors and mithramycin a impact a similar neuroprotective pathway at a crossroad between cancer and neurodegeneration. Pharmaceuticals.

[50] Choi M.C., Jong H.S., Kim T.Y., Song S.H., Lee D.S., Lee J.W., Kim T.Y., Kim N.K., Bang Y.J. (2004). AKAP12/Gravin is inactivated by epigenetic mechanism in human gastric carcinoma and shows growth suppressor activity. Oncogene.

[51] Choi M.C., Ryu S., Hao R., Wang B., Kapur M., Fan C.M., Yao T.P. (2014). HDAC4 promotes Pax7-dependent satellite cell activation and muscle regeneration. EMBO Rep..

[52] Choi M.C., Cohen T.J., Barrientos T., Wang B., Li M., Simmons B.J., Yang J.S., Cox G.A., Zhao Y., Yao T.P. (2012). A direct HDAC4-map kinase crosstalk activates muscle atrophy program. Mol. Cell.

[53] Ueta C., Iwamoto M., Kanatani N., Yoshida C., Liu Y., Enomoto-Iwamoto M., Ohmori T., Enomoto H., Nakata K., Takada K. (2001). Skeletal malformations caused by overexpression of CBFA1 or its dominant negative form in chondrocytes. J. Cell Biol..

[54] Glasson S.S., Chambers M.G., Van Den Berg W.B., Little C.B. (2010). The OARSI histopathology initiative—Recommendations for histological assessments of osteoarthritis in the mouse. Osteoarthr. Cartil..

