# Supplementary material for: Mithramycin A Alleviates Osteoarthritic Cartilage Destruction by Inhibiting HIF-2α Expression
Source: Int J Mol Sci. 2018 May 9;19(5):1411. doi: 10.3390/ijms19051411 (PMC5983647; doi:10.3390/ijms19051411)
Supplement: Supplementary file 1 [file ijms-19-01411-s001.pdf]

**Table S1.** The primers used for real-time PCR.

| Gene | Strand | Primer sequences | Product Size | Annealing Temp. |
|---|---|---|---|---|
| *Adamts4* | S | 5’-CATCCGAAACCCTGTCAACTTG-3’ | 281bp | 58°C |
| | AS | 5’-GCCCATCATCTTCCACAATAGC-3’ | | |
| *Adamts5* | S | 5’-GCCATTGTAATAACCCTGCACC-3’ | 292 bp | 58°C |
| | AS | 5’-TCAGTCCCATCCGTAACCTTTG-3’ | | |
| *Gapdh* | S | 5’-TCACTGCCACCCAGAAGAC-3’ | 450 bp | 58°C |
| | AS | 5’-TGTAGGCCATGAGGTCCAC-3 | | |
| *Epas1* | S | 5’-CGAGAAGAACGACGTGGTGTTC-3’ | 333 bp | 64°C |
| | AS | 5’-GTGAAGGCTGGCAGGCTCC-3’ | | |
| *Mmp2* | S | 5’-CCAACTACGATGATGAC-3’ | 233 bp | 60°C |
| | AS | 5’-ACCAGTGTCAGTATCAG-3’ | | |
| *Mmp3* | S | 5’-TCCTGATGTTGGTGGCTTCAG-3’ | 102 bp | 58°C |
| | AS | 5’-TGTCTTGGCAAATCCGGTGTA-3’ | | |
| *Mmp9* | S | 5’-ACCACATCGAACTTCGA-3’ | 212 bp | 58°C |
| | AS | 5’-CGACCATACAGATACTG-3’ | | |
| *Mmp12* | S | 5’-CCCAGAGGTCAAGATGGATG-3’ | 482 bp | 60°C |
| | AS | 5’-GGCTCCATAGAGGGACTGAA-3’ | | |
| *Mmp13* | S | 5’-TGATGGACCTTCTGGTCTTCTGG-3’ | 473 bp | 58°C |
| | AS | 5’-CATCCACATGGTTGGGAAGTTCT-3’ | | |
| *Mmp14* | S | 5’-GTGCCCTAGGCCTACATCCG-3’ | 580 bp | 62°C |
| | AS | 5’-TTGGGTATCCATCCATCACT-3’ | | |
| *Mtf1* | S | 5’-GTTTTAATGGTGATGCAGAGTCCGTC-3’ | 461 bp | 62°C |
| | AS | 5’-GGGATTATTAGTTAGGACAGAGTTGGC-3’ | | |
| *Sp1* | S | 5’-TGACAGGTCTCCCTGGAGTAAT-3’ | 476 bp | 58°C |
| | AS | 5’-GGAGCTGGAACTAGCTTGTGAT-3’ | | |
| *Zip8* | S | 5’-GAACAATTGCCTGGATGATCACGC-3’ | 430 bp | 62°C |
| | AS | 5’-AAGCCGGTTAACATCCCTGCATTC-3’ | | |
